# Anticancer Activity of Aqueous Extracts from *Asparagus officinalis* L. Byproduct on Breast Cancer Cells

**DOI:** 10.3390/molecules26216369

**Published:** 2021-10-21

**Authors:** Arianna Romani, Fabio Casciano, Claudia Stevanin, Annalisa Maietti, Paola Tedeschi, Paola Secchiero, Nicola Marchetti, Rebecca Voltan

**Affiliations:** 1 Department of Translational Medicine and LTTA Centre, University of Ferrara, 44121 Ferrara, Italy; arianna.romani@unife.it (A.R.); fabio.casciano@unife.it (F.C.); paola.secchiero@unife.it (P.S.); 2 Interdepartmental Research Center for the Study of Multiple Sclerosis and Inflammatory and Degenerative Diseases of the Nervous System, University of Ferrara, 44121 Ferrara, Italy; 3 Department of Chemistry, Pharmaceutical and Agricultural Sciences, University of Ferrara, 44121 Ferrara, Italy; claudia.stevanin@unife.it (C.S.); annalisa.maietti@unife.it (A.M.); paola.tedeschi@unife.it (P.T.); 4 Terra&Acqua Tech Lab, Ferrara Technopole, 44121 Ferrara, Italy

**Keywords:** *Asparagus officinalis*, breast cancer, cellular migration, reactive oxygen species (ROS)

## Abstract

Cultivation of asparagus (*Asparagus officinalis* L.; Asp) for food and medicinal use has taken place since the early Roman Empire. Today, Asp represents a worldwide diffuse perennial crop. Lower portions of the spears represent a food industry waste product that can be used to extract bioactive molecules. In this study, aqueous extracts derived from the non-edible portion of the plant (hard stem) were prepared and characterized for chemical content. Furthermore, the biocompatibility and bioactivity of Asp aqueous extracts were assessed in vitro on normal fibroblasts and on breast cancer cell lines. Results showed no interference with fibroblast viability, while a remarkable cytostatic concentration-dependent activity, with significant G1/S cell cycle arrest, was specifically observed in breast cancer cells without apoptosis induction. Asp extracts were also shown to significantly inhibit cell migration. Further analyses showed that Asp extracts were characterized by specific pro-oxidant activity against tumoral cells, and, importantly, that their combination with menadione resulted in a significant enhancement of oxidants production with respect to menadione alone in breast cancer cells but not in normal cells. This selectivity of action on tumoral cells, together with the easiness of their preparation, makes the aqueous Asp extracts very attractive for further investigation in breast cancer research, particularly to investigate their role as possible co-adjuvant agents of clinical drug therapies.

## 1. Introduction

Breast cancer (BC) is the most common cancer among women [1]. It is classified mainly based on the expression of three receptors that determine its pharmacological response and, therefore, severity: estrogen receptor (ER), progesterone receptor (PgR), and epidermal growth factor receptor 2 (HER2). The majority of cases are hormone-dependent, showing overexpression of ER and PgR [2]. In those patients, endocrine-based treatment is used as a first-line treatment; however, after its initial efficacy, about 50% of patients develop endocrine resistance and treatment failure, requiring different choices or use of combined therapy [3]. Triple-negative breast cancers (TNBC; ER-, PgR-, HER2-), on the other hand, account for 10–15% of cases and still do not have efficient treatment. Since TNBC show an unfavorable prognosis and hormone-dependent BC has a 5-year survival rate of 25%, it is important to find an efficient and relatively safe cure to improve patients’ survival [4]. 

Increasing attention in novel drug research has been focused on natural products as a source of compounds that exhibit few adverse effects [5,6]. One of the most common vegetables reported with therapeutic proprieties is asparagus, a plant nutritionally and commercially important belonging to the Asparagaceae family. Worldwide, more than 200 species have been identified [7], and among them, *Asparagus officinalis* L. (Asp) is the main species cultivated and commercialized [8]. The 2019 survey by FAO (http://www.fao.org/faostat/en/#data, on 5 October 2021 ) evidenced that asparagus cultivation is distributed worldwide, even if only six countries demonstrate major production: China (over 8 million tons); Peru (367,000 tons); Mexico (272,000 tons); Germany (131,000 tons); Spain (59,000 tons); Italy (50,000 tons). Italy has the largest yield in Europe and holds third place globally (7 tons per hectare), while the top yield globally belongs to Peru (11.5 tons per hectare). In Italy, asparagus production is focused only on five regions, with nearly 10,000 hectares cultivated (CSO Italy, Centro Servizi Ortofrutticoli, https://www.csoservizi.com/focus-prodotti/asparagi/, on 5 October 2021) by large agri-food farms. Hence, it is promptly understandable that potential solutions facing the recovery of bioactive molecules with asparagus waste material (i.e., hard stems, roots, plant aerial parts) might have a great impact on this agri-food chain.

Different factors, including species, type of cultivation, seasons, or plant section used greatly change the content of the active biological molecules extracted [9]. Previous studies, focused mainly on the stem portion, have identified phenolic compounds, saponins, sterols, sulfur-containing acid, carotenoid, and amino acids as the main bioactive components [10]. Components such as flavonoids and carotenoids are well known for their antioxidant activity; others, such as saponins, have been reported to possess cell cycle modulatory activity in cancer cells [11]. Lately, asparagus methanol extracts from *Asparagus laricinus* have been reported to induce cytotoxicity in breast and prostate cancer cell lines [12].

More recently, Asp has become of interest for the possibility to extract bioactive molecules from non-edible portions [13]. Indeed, besides edible stems used in industrial processing, hard stems and roots, which are normally discarded, have been suggested to be used to extract phytochemicals with biological activities [7,14]. Recycling food industry byproducts will be beneficial from both economic and ecological points of view. 

Therefore, the present study first had the purpose of characterizing the chemical composition of hard stem Asp extracts prepared by a simple aqueous method that does not alter the structure nor the biological activity of important functional molecules. Then, the next aim of the study was to investigate in vitro the biological effects of the water extracts on survival and proliferation, oxidative balance and migration of BC cell lines belonging to different phenotypes, as well as in a normal cell line. 

The simple procedure to obtain water extracts of Asp hard stem, its chemical composition and its bioactivity on breast cancer cell lines represent the most innovative key-points of this study. 

## 2. Results

### 2.1. Asparagus Extracts

Asparagus matrix has a moisture content of about 89.9% ± 0.1% *w*/*w*, as estimated by freeze-drying. The yield of water extraction was 7.25% ± 0.23% *w*/*w*. The estimation of total phenolic content (TPC), total flavonoid content (TFC) and antioxidant activity (DPPH and ABTS) for aqueous extract are reported in Table 1. TPC value evidenced a moderate amount of phenols extracted in Asp hard stem, confirmed also by low levels of antioxidant activity. As a matter of fact, the water extract contains 3.6% of phenolic compounds and 4% of flavonoids that constitute the hard stem matrix (see Table S1, Supplementary Material). It seems, therefore, that the aqueous extract is made of similar relative amounts of TPC and TFC than those available in ASP hard stem for extraction. Interestingly, flavonoids represent about 92.3% of water-extracted phenolic compounds, while in the hard stem, their relative amount is 83.8% of total phenolics on a dry matter basis. Thus, since phenolic compounds and flavonoids are water-extracted to the same extent (3.6% and 4%, respectively), and the relative amount of flavonoids is about 8.5% higher in dry water extracts than in dry hard stems, this can be interpreted in two ways: the dry water extract contains a slightly larger representative fraction of flavonoids than ASP hard stem matrix; or part of the water-extracted phenolics are lost during the lyophilization process and the relative amount of flavonoids results a bit higher. 

### 2.2. LC-MS/MS Analysis of Asparagus Extracts 

#### 2.2.1. Free Amino Acid Profile

Proteins and peptides in the hard stem represent about 1.85% *w*/*w* on a fresh matter basis and exhibit antioxidant and ACE inhibitory properties, as already evidenced recently [15]. Here, we mainly focused on the free aminoacidic profile. Single amino acid concentrations are reported in Table 2. Ion transitions (i.e., MRM (*m*/*z*) column) allowed us to correctly identify all amino acids, except for leucine (LEU) and Isoleucine (ILE). These two were quantified together. The total amount of free amino acids in the hard stem is noticeably higher (i.e., 45 μg/mg_de_) than in the edible stem (i.e., 32.7 μg/mg_de_) (data not published). The most abundant free amino acids in the hard stem are lysine (56.3%) and asparagine (30.3%). Proline, alanine, valine and glutamic acid are in the range 1–3%. Arginine, aspartic acid, histidine, leucine and isoleucine, methionine, phenylalanine, serine, threonine and tryptophan are in the range 0.1–0.9%, while only tyrosine is below 0.1%. Tryptophane is about 35 times more concentrated in the hard stem than in the edible part of the stem, while histidine and arginine are only 1.9 and 1.5 times higher in the hard stem. Additionally, levels of lysine and asparagine are higher in the hard stem than in the whole edible stem and spear: even though they are still the most abundant amino acids in the edible part, lysine is about 1.4 times and asparagine is 1.7 times more abundant in the hard stem (see Table S2, Supplementary Material, for the free amino acidic profile in the edible stem).

#### 2.2.2. Organic Acids, Phenolic Acids, Polyphenols and Derivatives

Few secondary metabolites were identified in water extracts. Their tentative LC-MS/MS identification is reported in Table 3. Compounds are grouped based on their most intense ionization mode. The second column presents the precursor ion, while the third column contains all informative fragment ions useful for compound identification. Numbers in parenthesis are the ion relative intensity. The last column reports the relative abundance for each identified compound, calculated on the basis of peak area.

Among common organic acids (i.e., malic and quinic) and phenolic acid esters (i.e., dicaffeoyltartaric acid, caftaric acid, caffeoylquinic acid, feruloylquinic acid), important free aglycones were found: three flavonols (i.e., quercetin, kaempferol and syringetin), two isoflavones (i.e., genistein and prunetin) and one flavone (i.e., apigenin). Additionally, some common and less common glycosylated flavonoids were identified, such as myricetin-O-rhamnoside, tricin-O-glucoside, isorhamnetin-O-glucuronide, laricitrin-O-glucoside and rutin. Interestingly, two anthocyanins were identified (i.e., petunidin- and malvidin-O-pentoside derivatives) and listed separately as [M]^+^.

Both isoflavones and flavonols are known to play important bioactive roles as estrogens and antioxidants. Hence, alone or in synergy with all compounds identified in water extracts, they may have captivating effects on cancer cells. 

Compounds tentatively identified in Asp hard stem aqueous extracts are in accordance with literature works, although most of them concerned more exhaustive hydro-alcoholic extracts and most of the extensive chemical characterization regards the edible part [11,16,17,18,19,20].

### 2.3. Asparagus Extracts Affect Viability of MCF-7 Breast Cancer Cells through Cell Cycle Block

**Figure 1:**
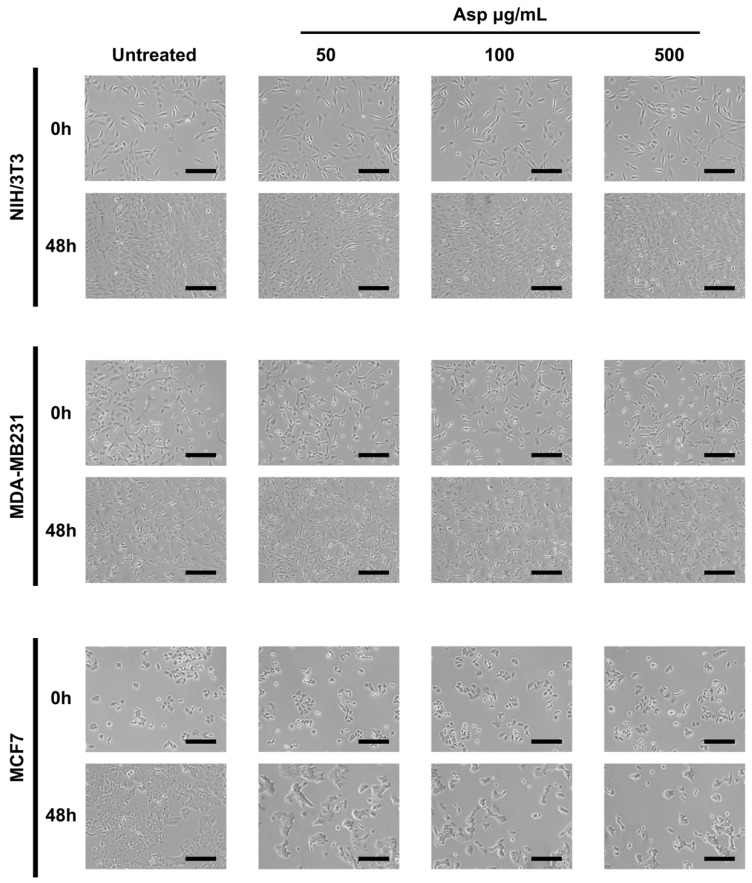
Representative images were taken by light microscopy of NIH/3T3, MDA-MB231 and MCF7 cells untreated or treated with 50, 100, and 500 µg/mL of Asparagus extracts (Asp) for 48 h. Magnification 10×, scale bar 200 µm.

**Figure 2:**
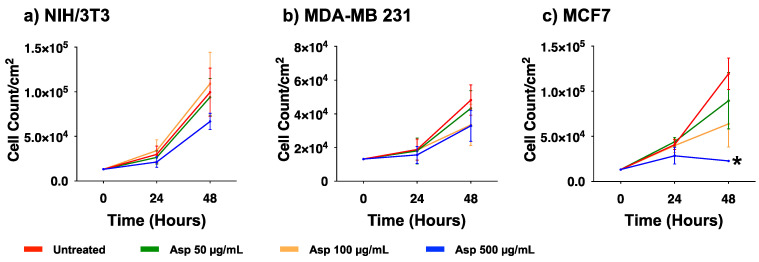
Cell viability of (**a**) NIH/3T3, (**b**) MDA-MB231, (**c**) MCF7 exposed to 50, 100, and 500 µg/mL of Asp for 24 and 48 h. Data are reported as mean ± standard error of the mean from three independent experiments. Statistical analysis was performed by ANOVA followed by Bonferroni’s post hoc test. * *p* ≤ 0.05 vs. untreated at the same incubation time.

To evaluate the biological effects of Asp extracts on cellular activities, cellular morphology, replication and apoptosis were assessed on NIH/3T3 fibroblasts and breast cancer cell lines MDA-MB231 and MCF7, before and after treatments with several doses of Asp extracts. As shown in Figure 1, no changes in morphology and distribution were observed in NIH/3T3 as well as in MDA-MB231 cells until 48 h after treatments. However, a remarkable reduction in cell density coupled with cell size increase (typical of cellular senescence) was observed in MCF7 cells treated for 48 h with Asp with respect to the untreated. In agreement, as reported in the panels in Figure 2a,b, no significant changes in the number of viable cells were identified in NIH/3T3 and MDA-MB231 cells. On the contrary, a significant reduction in the number of viable cells was identified in the MCF7 cell line treated with 500 µg/mL Asp extracts for 48 h, compared with untreated cells maintained in culture in the same condition (Figure 2c; *p*-value 0.0434).

**Figure 3:**
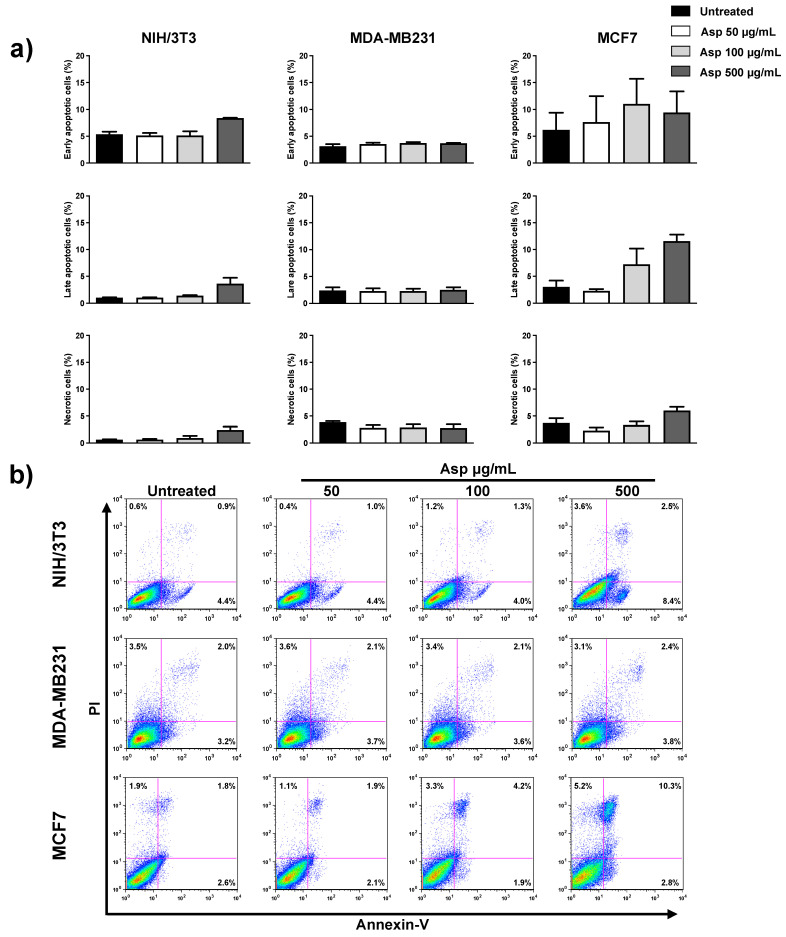
NIH/3T3; MDA-MB231; MCF7 percentage of apoptotic cells after 48 h, calculated from the flow cytometry dot plots after Annexin V/PI staining. (**a**) Results from untreated or treated with 50, 100, and 500 µg/mL Asp extracts were expressed as a percentage of the total population. Data are reported as mean ± standard error from three independent experiments. (**b**) Representative flow cytometry dot plots. The axis scales for fluorescence are reported as logarithmic.

Next, we investigated whether induction of apoptosis was responsible for the observed effects on cell viability. As reported in Figure 3, there were no significant differences between treated and untreated samples for all three cell lines, analyzing early and late apoptotic events, as well as necrotic events. Only for the MCF7 cell line, an increasing trend was observed for late apoptotic population in response to increasing concentrations of extracts, even if it was not significant with respect to the untreated control. 

**Figure 4:**
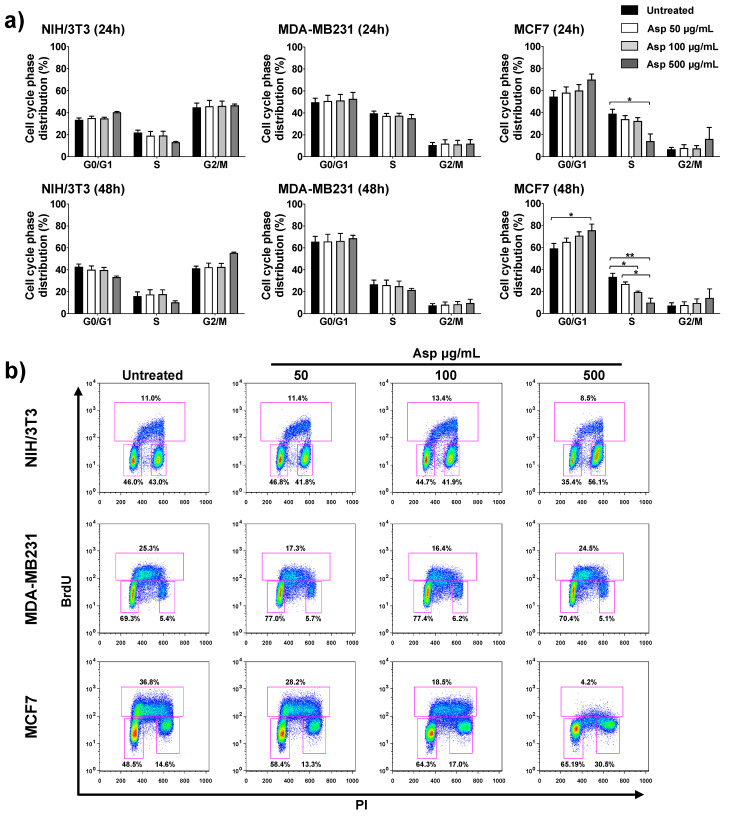
NIH/3T3; MDA-MB231; MCF7 cell distribution in the different phases of the cell cycle calculated from the flow cytometry dot plots after BrdU/PI staining of cultures after 24 or 48 h. (**a**) Results from cells untreated or treated with 50, 100, and 500 µg/mL of Asp extracts were expressed as a percentage of the total population. Data are reported as mean ± standard error from three independent experiments. Statistical analysis was performed by ANOVA followed by Bonferroni’s post hoc test. *p*-values ≤ 0.05 were considered significant: * *p* ≤ 0.05, ** *p* ≤ 0.01; (**b**) Representative flow cytometry dot plots of cell cycle profiles after 48 h of treatments. The PI axes scale is reported as linear and the BrdU axes scale is reported as logarithmic.

To further investigate the possible implication of Asp extracts on cell proliferation, the cell cycle was analyzed by flow cytometry upon BrdU incorporation. In agreement with cell viability results, Asp treatment on NIH/3T3 and MDA-MB-231 cells did not modify the cell cycle phase distribution either after 24 h or 48 h of treatment (Figure 4). By contrast, in MCF7 cells, Asp (500 µg/mL) induced a relevant contraction of the number of cells in S phase already after 24 h, when compared with untreated (*p*-value = 0.0169). Extending the treatment to 48 h, the alteration of cell cycle phases was more marked, showing a concentration-dependent effect with a significant reduction in cells in the S phase (100 µg/mL vs. untreated, *p*-value = 0.0469; 500 µg/mL vs. untreated, *p*-value = 0.0016; Asp 50 µg/mL vs. Asp 500 µg/mL, *p*-value = 0.0205), accumulation in G1 (500 µg/mL vs. untreated, *p*-value= 0.0169) and consequent induction of cell cycle blockade.

### 2.4. Asparagus Extracts Cause ROS Production in Breast Cancer Cells but Not in Normal Cells

To further study the biological effects of Asp extracts in normal (NIH/3T3) and tumoral (MCF7) cell lines, a possible role of Asp in modulating the production of oxidants upon treatment was assessed by a method that enables the flow cytometric detection of reactive oxygen species (ROS) in live cells (CellROX Green Flow Cytometry Assay).

**Figure 5:**
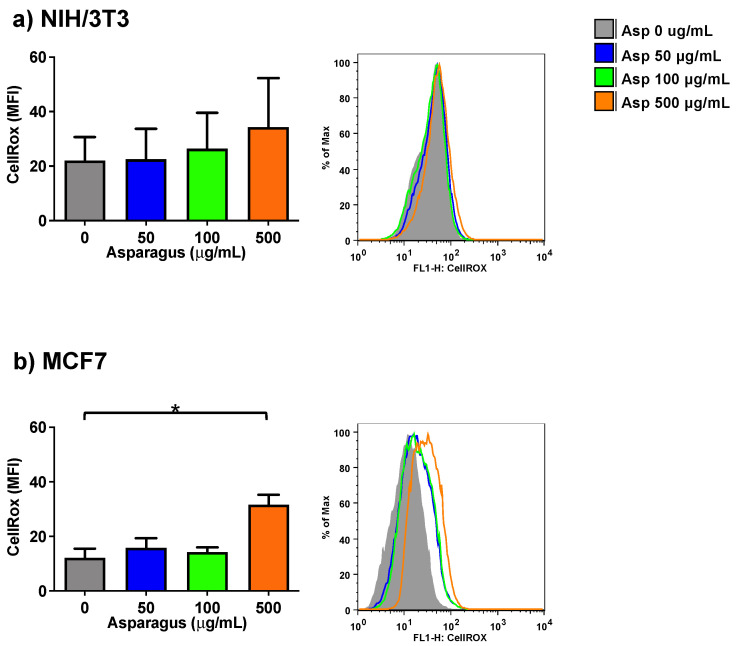
NIH-3T3 (**a**) or MCF7 (**b**) cells were treated with 0, 50, 100 or 500 µg/mL of Asp extracts for 48 h and incubated with CellROX Green Reagent to investigate the oxidation level (stress) of the cells. Left panels: MFI data are reported as mean ± standard error of results from three independent experiments; right panels: super imposed graphs from flow cytometry dot plots data of a representative experiment. The axes scale for fluorescence is reported as logarithmic. Statistical analyses were performed by ANOVA followed by Bonferroni’s post hoc test. * *p* < 0.05.

As depicted in Figure 5, following exposure to several Asp concentrations, normal cells did not significantly change their ROS level, while a significant increase in the production of oxidant species was observed in breast cancer MCF7 cells treated with 500 µg/mL of Asp for 48 h, with respect to the untreated sample (Asp 0 µg/mL) (*p*-value = 0.0126).

### 2.5. Asparagus Extracts Increase Oxidant Activity of Menadione in Breast Cancer Cells

**Figure 6:**
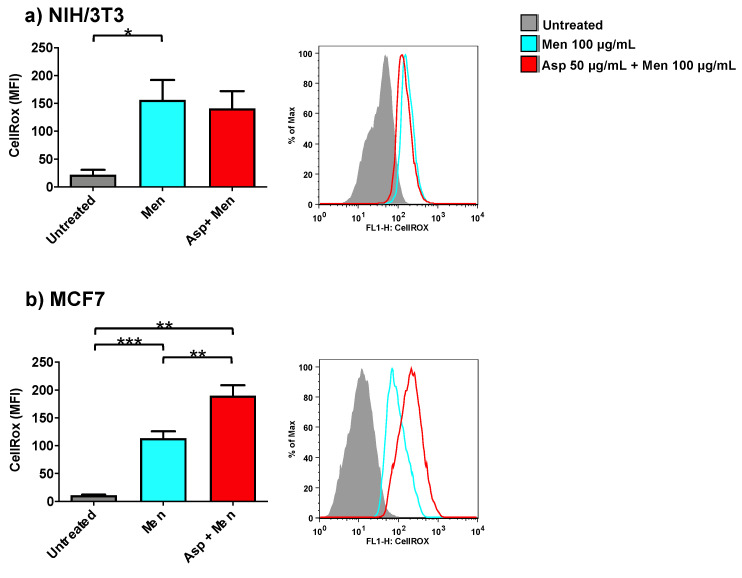
NIH/3T3 (**a**) or MCF7 (**b**) were treated with 50 µg/mL Asp for 48 h and then triggered with oxidative stimulation performed by Menadione (Men) 100 µM for 30 min. The left panels reported data as mean ± standard error of results from at least three independent experiments. Right panels: representative flow cytometry dot plots. The axes scale for fluorescence is reported as logarithmic. Statistical analysis was performed by ANOVA followed by Bonferroni post hoc test; * *p* < 0.05, ** *p* < 0.01 and *** *p* < 0.001.

In order to investigate the role of Asp extracts on oxidative balance, both normal (NIH/3T3) as well as tumoral cells (MCF7) were exposed to Asp and then triggered with a pro-oxidant concentration of menadione (Figure 6). Asp 50 µg/mL was chosen as the pre-treatment dose to exclude concomitant effects derived from an alteration in viability or cell cycle modulation.

As expected, cells treated only with Menadione (positive control) showed a significant increase in oxidant production compared to untreated cells (*p*-value 0.0425 and *p*-value ≤ 0.0001 for NIH/3T3 and MCF7, respectively). Interestingly, cells pre-treated with Asp extracts showed an opposite response in normal and cancerous cells when triggered with Menadione. Indeed, in fibroblasts (Figure 6a), Asp did not induce a significant variation with respect to Menadione; however, in MCF7 cells (Figure 6b), a combination of Asp and Menadione significantly enhanced oxidant production compared to cells treated with Menadione alone (*p*-value = 0.0038).

### 2.6. Asparagus Extracts Impair Cell Migration

**Figure 7:**
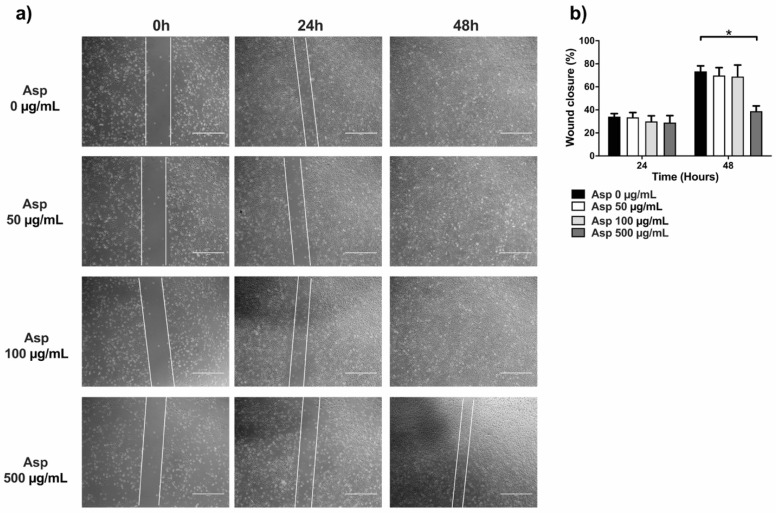
Scratch assay performed on fibroblasts (NIH/3T3). (**a**) Representative images were taken under light microscopy of fibroblast monolayers untreated or treated with 50, 100, and 500 µg/mL of asparagus (Asp) at 0 h (immediately after scratch were performed), 24 or 48 h. Scale bar 1000 µm. (**b**) Data from four independent experiments are reported as mean ± standard error. Statistical analyses were performed by ANOVA followed by Bonferroni’s post hoc test. * *p* < 0.05.

To complete the characterization of biological activities of Asp on cells, we first assessed effects on migration by using traditional wound repair. A single scratch was manually performed on NIH/3T3 cells grown with a reduced concentration of serum (5% FBS) to lower interference from normal cell replication. Cells were treated with Asp at different doses and pictures were taken at different time points until 48 h of incubation (Figure 7). At 24 h, no significant variation in wound area was found, although a significant reduction in wound closure was observed in fibroblasts treated with 500 µg/mL of Asp compared with untreated cells (Asp 0 µg/mL) after 48 h of treatment (*p*-value = 0.0103). 

**Figure 8:**
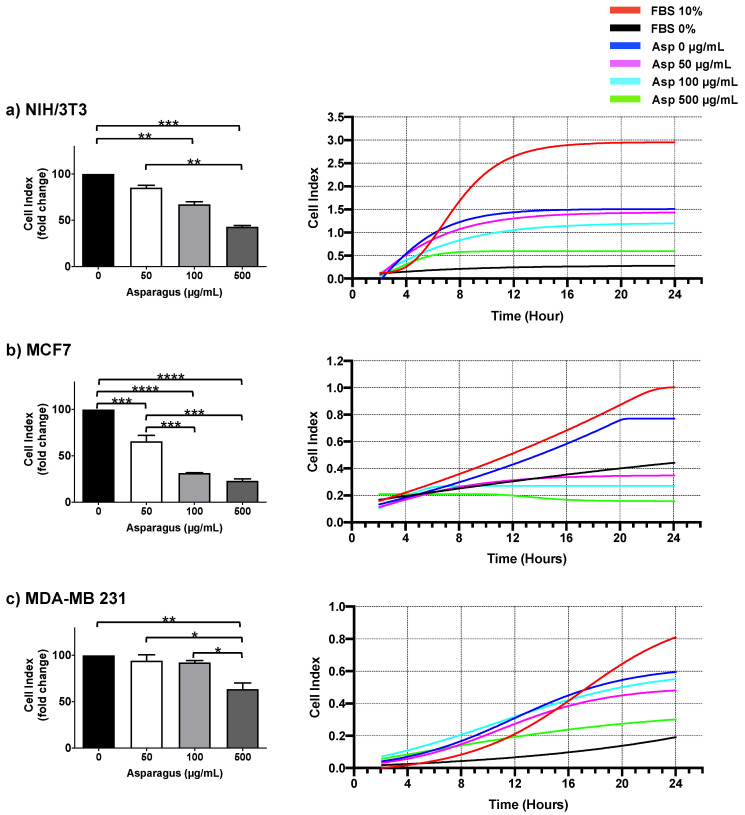
NIH-3T3 (**a**), MCF7 (**b**) and MDA-MB231 (**c**) cells were seeded in the upper chamber of CIM plate and monitored for migration through the lower chamber in response to different concentrations of Asp (0–500 µg/mL) with the xCELLigence system (DP-RTCA) for 24 h. Positive controls were performed by using medium with 10% FBS in the lower chamber as the chemoattractant, while negative controls were performed by using medium without FBS (0% FBS). In the left panels, data are expressed as fold change compared to untreated cells of results from three independent experiments and are reported as mean ± standard error. Statistical analysis was performed by ANOVA followed by Bonferroni’s post hoc test. *p*-values ≤ 0.05 were considered significant: * *p* ≤ 0.05, ** *p* ≤ 0.005, *** *p* ≤ 0.001 **** *p* ≤ 0.0001. Right panels show representative graphs from RTCA software reporting overtime cell index.

To deeply investigate the role of Asp extracts in cell motility, dynamic high-resolution assessments of migration using the xCELLigence assay were performed on both normal and breast cancer cells. Results showed a significant inhibitory effect on cell migration mediated by Asp extracts, with respect to untreated, in all cell line tested (Figure 8). In detail, a significant reduction in NIH/3T3 cell migration was observed in response to 100 and 500 µg/mL (*p*-value 0.0068 and ≤0.0001, respectively), confirming data from wound repair. Importantly, MCF7 cell lines showed a significant reduction in migration in a concentration-dependent manner (*p*-value ≤ 0.0001 for all dosed vs. untreated; *p*-value ≤ 0.001 for 100 µg/mL and 500 µg/mL vs. 50 µg/mL), reducing the migration of this cell line below the basal threshold (0% FBS). Of note, migration was also significantly impaired in triple-negative MDA-MB231 cells after treatment with Asp 500 µg/mL (*p*-value 0.0038 vs. untreated; *p*-value 0.0115 vs. Asp 50 µg/mL; *p*-value 0.0162 vs. Asp 100 µg/mL). 

## 3. Discussion

In recent years, increasing attention has been paid to sustainable recycling of food waste, mainly focusing on recovering bioactive molecules. This might represent a new source for drug production [21], especially for fields such as cancer where drug resistance or side effects still cause treatment interruption or failure. In the present study, we characterized the chemical composition and biological activities of hard stem asparagus aqueous extracts obtained from food industry waste recycling. 

The chemical composition analyses revealed the absence of many antioxidants that were frequently found on edible stems of asparagus. However, the presence of isoflavones, flavonols and flavones together with other glycosylated flavonoids might result in an effective pool of molecules with bioactivity on breast cancer cells. In particular, a relevant feature is represented by the presence of two isoflavones (i.e., prunetin and genistein) and one flavone (i.e., apigenin), counting all three together for the 1.7% relative amount in the extracts. It is known that these compounds might exert some antiestrogenic activity on estrogen-receptor-α positive tumor cells, such as MCF7. 

Indeed, we demonstrated in vitro, on cellular models of breast cancer with different phenotypes, specific anticancer effects, mainly attributable to cell cycle arrest, migration inhibition and oxidant production. 

Firstly, we observed a significant reduction in the proliferation rate of the MCF7 breast cancer cell line (expressing HR^+^/HER2^−^ phenotype), associated with a significant blockade of the cell cycle and low levels of apoptosis. This evidence is in accordance with other reports demonstrating cell cycle arrest in different cancer cell types following treatment with asparagus extracts [11,12]. However, studies are difficult to compare, since they use different raw materials and different methods for the extraction. We believe that the antiproliferative effect of Asp extracts on these cells might be the final result due to the combined effects of antiestrogenic phytocompounds and other antioxidants, mainly rutin, which is the most abundant flavonoid (i.e., 2.4%). Undoubtedly, the copresence of different components with activities connected to antitumor effects (i.e., anti-inflammatory), together with the antioxidant one (see rutin), might end up with particularly efficacious bioactivity against hormone-sensitive tumor cells. On the contrary, no direct involvement in these types of effects concerns the two organic acids (i.e., malic and quinic acids), which are the most abundant compounds in the extracts (i.e., 49% and 38%, respectively) and they are known to have antibacterial and anticholinesterase properties. Nonetheless, it is possible that malic and quinic acids can contribute somehow to the overall antioxidant property of the extract, as well as kaempferol and the other glycosylated flavonoids. Instead, three phenolic acids, such as caftaric (6.7%), dicaffeoyltartaric (0.5%) and caffeoylquinic (0.3%), can play a supportive role in affecting the tumor cell cycle and its response to other compounds.

Our results also indicated that any significant changes in proliferation, cell cycle and apoptosis were observed in MDA-MB-231 triple-negative cells (representative of a more aggressive phenotype of breast cancer), but not in NIH/3T3 noncancer cells. 

Phytochemicals are commonly associated with beneficial effects, mainly for the presence of antioxidant molecules; however, the asparagus extracts used in our study, derived from the portion discarded from the food industry, were characterized by poor antioxidant content. This apparent defect translated into an advantage when we assessed the extracts on MCF7 tumoral cells. Indeed, in this model, we demonstrated a specific significant pro-oxidant activity of the extracts used alone at high dosage and, importantly, their capability at low dosage to enhance the level of ROS production induced by menadione. To explain this last feature, we can assume that the antioxidant molecules, even if present at low levels in the extracts, might be sufficient to induce redox amplification, cooperating in the ROS production prompted by menadione. This concept is supported by other authors that previously demonstrated an interaction between menadione and antioxidant molecules, resulting in increased oxidative stress in the same model—the MCF7 cells [22]. They reported apoptosis induction by ascorbate/menadione combination treatment, suggesting inhibition of ERK2 induced by H_2_O_2_ generated from menadione redox cycling, with specific action on cancer cells. Our results similarly showed a specific cooperative effect on HR^+^/HER2^−^ cells (MCF7), while preserving normal control fibroblasts. To further support our theory, it is of interest to cite Bakalova et al., who demonstrated that using menadione/ascorbate in combination with 13 conventional clinically used drugs for leukemia treatment did not induce cytotoxicity in cancer cells, but instead, caused irreversible metabolic changes, increasing sensitivity toward conventional therapy [23]. These observations are particularly important because they lay the foundations for future use of extracts to enhance the effects of clinically used drugs that act on breast cancer cells through the induction of oxidative stress, such as tamoxifen (used for hormonal therapy) or paclitaxel (used for chemotherapy). This aspect deserves further preclinical studies to evaluate its feasibility and effectiveness. 

Next, we observed a significant inhibition of concentration-dependent migration of MCF7 cells and, surprisingly, even of the triple negative MDA-MB-231 cells. In particular, for MCF7, which showed a low degree of apoptosis after treatment with ASP, we exclude the role of cell death due to the intrinsic characteristics of the assay that allows seeding of the cells in the upper chamber, while the medium with treatment is placed only in the lower chamber. Inhibition of cell migration on MDA-MB-231 cell models has been described following treatments with traditional anticancer drugs (e.g., cisplatin) [24] and purified molecules (e.g., curcumin) [25]. Interference with cellular migration represents a pivotal effect of the anticancer activity of the extracts, suggesting a possible role in inhibition of the metastatic process. A separate explanation is needed to interpret the data obtained on the migration of NIH/3T3 noncancer cells. In fact, these cells are fibroblasts and, as cells normally present in the connective tissue, in the case of tumors, they become part of the tumor microenvironment. Here, they are known to release substances that promote tumor proliferation [26]. In addition, they are also able to migrate to tumor implantation sites (or metastases) to support their growth through the induction of angiogenesis [27]. Therefore, inhibiting the migration of these cells, even if of the nontumor phenotype, has important implications in preventing the development of a tumor-friendly microenvironment.

This is a preliminary study that will need more investigations to comprehend the molecular mechanisms involved in the observed biological effects, as well as to understand the efficacy of the combined use of Asp extracts with traditional drugs by using preclinical in vitro and in vivo models. Nonetheless, we believe that our results will open up new opportunities to use natural products, alone or combined with traditional treatment, to improve therapy for BC. In particular, observations reporting the inhibition of proliferation and menadione combined oxidant production in HR^+^ cells, as well as migration reduction in both HR^+^ and TNBC cells, seem to be particularly relevant for patients who develop drug resistance.

## 4. Materials and Methods

### 4.1. Preparation of Asparagus Extracts, Phenolic Content, Total Flavonoids and Antioxidant Activity

Asp hard stem samples employed in this study were from a farm located in the Province of Rovigo (Veneto Region, Italy). They were waste materials from greenhouse cultivation of Asp obtained when harvested spears are sorted and packed for the market. 

Aqueous extracts from lyophilized Asp hard stems were obtained by the following procedure, which has already been applied with modifications to other vegetable matrices [15,28,29,30]: ultraturrax (T18 model by IKA-Werke, Staufen, Germany) mechanical homogenization of 1 gram (dry matrix) in 25 mL of water (1:25 *w*/*v*) for 1 min; ultrasound bath (Elmasonic S 30 H, Elma Schmidbauer, Singen, Germany) for 10 min at 37 kHz (80 W effective ultrasonic power); suspension centrifuged at 10,000 g for 10 min (model PK121R, Thermo Fisher Scientific, Waltham, MA, USA). Then, water supernatants were recovered, and the extract was lyophilized. 

Phenolic content and antioxidant activity were determined by means of four different parameters: total phenolic compounds (TPC); total flavonoids (TFC); two Trolox equivalent antioxidant capacity (TEAC) assays based on DPPH and ABTS radicals. TPC and TFC are reported as μg of gallic acid equivalents per mg of dry extract (μg_GAE_/mg_de_) and they were determined in accordance with Singleton [31] and the aluminum chloride colorimetric method [32,33], respectively. Results for DPPH and ABTS assays were calculated as μmol of Trolox equivalents per mg of dry extract (μmol_TE_/mg_de_).

These parameters were also estimated for hard stems (on dry basis) by using an overnight hydroalcoholic extraction (80:20 methanol:water + 0.1% *v*/*v* of formic acid) [25]. They were similarly expressed as μg_GAE_/mg_dm_ and μmol_TE_/mg_dm_, where mg_dm_ means mg of dry matter (i.e., lyophilized ASP byproducts).

### 4.2. LC-MS/MS Analysis of Asparagus Extracts

#### 4.2.1. Free Amino Acid Profile

Protein content and bioactive peptides in this byproduct matrix have already been investigated by the same authors [14]. Thus, here, we focused more on the determination of free amino acids. They were determined by external calibration method. Liquid chromatographic separation was obtained on a Restek Raptor Polar X column (100 × 2.1 mm) packed with superficially porous particles (diameter 2.7 μm). The mobile phase was a mixture of water + formic acid 0.1% (*v*/*v*) (A) and acetonitrile + formic acid 0.1% (*v*/*v*) (B). Chromatographic runs were conducted under gradient elution conditions (from 25% to 70% of A in 10 min) at a mobile phase flow rate of 150 μL/min.

MS/MS detection was carried out under MRM mode (multiple reaction monitoring) with the positive ESI mode (see Section 2.2.1 for ion transitions and results). 

#### 4.2.2. Organic Acids, Phenolic Acids, Polyphenols and Derivatives

Untargeted LC-MS/MS analysis of phenolics was carried out by means of a 2.1 × 150 mm Symmetry C18 column (Waters, Milford, MA, USA), packed with 3.0 mm fully porous particles, thermostated at 30 °C, under gradient elution conditions. Mobile phases were mixtures of water and formic acid 0.1% (*v*/*v*), channel A, and acetonitrile and formic acid 0.1% (*v*/*v*), channel B. The gradient elution was obtained with 5% to 70% channel B in 25 min. MS detection was achieved with a Thermo LTQ XL linear ion trap, equipped with an ESI interface (both positive and negative ionization modes were used). 

### 4.3. Cell Culture and Treatment

Mouse embryo fibroblasts (NIH/3T3) and human breast cancer cell lines MDA-MB-231 (TNBC) and MCF7 (ER^+^, HER2^−^) were purchased from Lonza (Basel, Switzerland) and grown in DMEM (4.5 g/L glucose) containing 10% FBS, 1% pen/step and L-glut (all from Life Technologies, Monza, Italy). Cells were maintained at 37 °C in a humidified atmosphere with 5% CO_2_. Doubling times of the three cell lines were determined by the calculator available at http://www.doubling-time.com/compute.php on 28 September 2021 (Roth V. 2006 Doubling Time Computing; NIH/3T3: 15 ± 1 h; MCF7: 15 ± 2 h; MDA-MB231: 22 ± 2 h). Lyophilized Asp extracts were dissolved in DMEM containing 1% FBS, 1% pen/strep, and L-glut, sterilized by filtration and used for in vitro treatment at predetermined concentrations of 50, 100, and 500 µg/mL for 24 and 48 h on NIH/3T3, MDA-MB231 and MCF7 cells seeded at the density of 25,000 cells/cm^2^. Cells were analyzed for cell shape and growth changes. Phase-contrast images were recorded with an EVOS digital microscope (Advanced Microscopy Group, Bothell, WA, USA). Recorded images were normalized for brightness with Fiji software [34].

### 4.4. Assessment of Cell Viability, Cell Cycle Profile and Apoptosis

Twenty-four and 48 hours after treatment with Asp extracts, cell viability was examined by Trypan blue exclusion dye. Cell cycle profiles were analyzed by 5-bromodeoxyuridine (BrdU; Sigma, St Louis, MO, USA) incorporation, as previously described [35]. Briefly, cells after incubation with 50 µM BrdU for 1 h at 37 °C were stained with primary mouse anti-BrdU antibody (clone 3D4, BD Bioscience, San Josè, CA, USA), goat F(ab’)2 anti-mouse IgG (H+L) fluorescein isothiocyanate-conjugated secondary antibody (Beckman Coulter, Brea, CA) and propidium iodide (PI; Sigma, St Louis, MO, USA), and were acquired using an FACS Calibur flow cytometer (BD Bioscience, San Josè, CA, USA) [36]. Apoptosis was analyzed in flow cytometry by Annexin V-FITC/propidium iodide (PI) staining (Immunotech, Marseille, France), as previously reported [36]. Flow cytometric acquisition was analyzed with the FlowJo software (Tree Star, Ashland, OR, USA).

### 4.5. Analysis of ROS 

Changes in intracellular reactive oxygen species (ROS) levels were determined by cell-permeable CellRox^®^ Green Oxidative Stress Reagent (Molecular Probes, Life Technologies) and analyzed using FACS Calibur flow cytometer. CellRox^®^ is a nonfluorescent (or very weak fluorescent) dye in a reduced state, which is used for ROS measurement in live cells based on the strong fluorogenic signal developed after oxidation. Briefly, 50,000 NIH/3T3 or MCF7 cells were seeded on 24-well plates and grown for 24 h. The next day, the medium was removed, and cells were treated with 50 µg/mL of Asp extracts (diluted in fresh medium) for 48 h or left untreated as a negative control. As a positive ROS control, cells were treated with 100 µM Menadione (Sigma-Aldrich, St Louis, MO, USA) for 30 min. Cells were then stained with 2.5 µM CellRox^®^ reagent for 30 min at 37 °C protected from light. 

Cells were harvested by trypsinization, washed with PBS and centrifuged at 125*xg* for 5 min. In the end, cells were re-suspended in PBS with 0.5 µg/mL 7-AAD (BD Bioscience, San Josè, CA, USA) and analyzed by flow cytometry. Dead cells, positive for 7-AAD staining, were excluded from the analysis. Flow cytometric acquisition was analyzed with the FlowJo software (Tree Star, Ash-land, OR, USA).

In some experiments, 100 µM menadione was added for 30 min to NIH/3T3 or MCF7 pretreated with 50 µM Asp for 48 h and then stained with 2.5 µM CellRox^®^ reagent for 30 min at 37 °C protected from light. Flow cytometric acquisition was performed as reported above.

### 4.6. Wound Healing Assay

NIH-3T3 cells were seeded on a 6-well plate (13,000 cell/cm^2^) in DMEM, 5% FBS, 1% sodium pyruvate, 1% pen/step and L-glut and left to grow for 24 h. A linear scratch was performed in the middle of each well using a p200 tip; then, the medium was removed and cells were washed twice with PBS before adding fresh media containing a predetermined concentration of 5% FBS and Asp extracts (0, 50, 100 or 500 µg/mL). Phase-contrast images were recorded with an EVOS digital microscope. Four frames for each well were taken following the scratch length at different time points (immediately after the scratch was performed, after 24 and 48 h). For each well, analysis of the scratched area was performed by the Fiji software edge recognition tool and calculated by the following formula:

$$\mathrm{Wound}\;\mathrm{closure}\;(\%)=⎡\frac{A_{\mathrm{to}}-A_{\Delta t}}{A_{t0}}⎤\times 100$$

where $A_{\mathrm{to}}$ is the average of four frames measured immediately after scratching and A_∆t_ is the average of four frames measured after 24 or 48 h after scratching. Wound closure in cells treated with Asp extracts was compared with untreated cells at the same time point. 

### 4.7. Assessment of Cell Migration

Cell migrations were performed using a DP-RTCA xCELLigence real-time cell analyzer (F. Hoffmann-La Roche SA, Basel, Switzerland) which records changes in impedance (reported as Cell Index) over time in a non-invasive system. Migration assay was performed using RTCA DP CIM-Plates 16. Cells were starved for 2 h with DMEM containing 0.2% FBS, 1% pen/strep, and L-glut and then seeded (40,000 cells/well) in the upper chamber in DMEM containing 1% FBS, 1% pen/strep, and L-glut. A 1% FBS concentration was experimentally established to support the length (24 h) of the experiments. Each condition was run in triplicate. Cells were left to equilibrate at RT for 30 min. Migration kinetics were analyzed in the presence of Asp (0, 50, 100, 500 µg/mL in DMEM containing 1% FBS, 1% pen/strep, and L-glut) in the lower chamber and recorded every 15 min for 48 h. The positive control was performed using DMEM containing 10% FBS in the lower chamber as the chemoattractant. Data were analyzed using the RTCA software (F. Hoffmann-La Roche, version 1.2.1, Basel, Switzerland) and expressed as fold change ± SEM of CI of migration with respect to untreated cultured set.

### 4.8. Statistical Analysis

All data, obtained from independent experiments, were tested for normal distribution by the Shapiro–Wilk normality test and for homogeneity of variance by the Brown–Forsythe test. Results were evaluated by one-way ANOVA followed by Bonferroni post hoc test (from multiple corrections) using GraphPad Prism (GraphPad Software, San Diego, CA, USA). Results were expressed as mean ± standard error of the mean (SEM) of replicate experiments. Statistical significance was defined as *p* < 0.05.

## Figures and Tables

**Table 1:** Values of total phenolic content (TPC), total flavonoid content (TFC) and antioxidant activities (DPPH and ABTS) in water extracts from asparagus hard stem byproducts. Values are expressed in terms of gallic acid equivalents (GAE) and Trolox equivalents (TE) per mg of dry extract (mg_de_).

**TPC** (μg_GAE_/mg_de_)	10.09 ± 1.23
**TFC** (μg_GAE_/mg_de_)	9.31 ± 0.74
**DPPH** (μmol_TE_/mg_de_)	0.010 ± 0.006
**ABTS** (μmol_TE_/mg_de_)	0.017 ± 0.006

**Table 2:** Quantitative determination of free amino acids in asparagus hard stem byproducts.

Amino Acid	Concentration (μg/mg_de_)	MRM (*m*/*z*)
ALA	0.101 ± 0.023	90.2 → 44.2
ARG	0.369 ± 0.019	175.1 → 70.1
ASN	13.65 ± 1.12	133.1 → 74.1
ASP	0.322 ± 0.022	134.1 → 74.1
GLU	0.451 ± 0.027	148.2 → 84.2
HIS	0.254 ± 0.015	156.1 → 110.2
LEU+ILE	0.368 ± 0.018	132.1 → 86.1
LYS	25.33 ± 1.75	147.1 → 84.1
MET	0.069 ± 0.004	150.2 → 104.1
PHE	0.117 ± 0.012	166.2 → 120.2
PRO	1.434 ± 0.068	116.1 → 70.1
SER	0.364 ± 0.016	106.1 → 60.1
THR	0.253 ± 0.011	120.1 → 74.1
TRP	0.144 ± 0.009	205.2 → 146.2
TYR	0.039 ± 0.007	182.2 → 136.2
VAL	0.814 ± 0.034	118.1 → 72.1

**Table 3:** Tentative LC-MS/MS identification of most abundant compounds in asparagus hard stem.

Compound	[M − H]^−^ (*m*/*z*)	MS/MS Fragments (*m*/*z*)	Relative Abundance
Dicaffeoyltartaric acid	473	311 (100), 293 (80) 179 (5), 149 (3)	0.5%
Caftaric acid	311	149 (100), 179 (55), 135 (5)	6.7%
Caffeoylquinic acid	353	191 (100), 179 (5)	0.27%
Malic acid	133	115 (100)	48.8%
Myricetin-O-rhamnoside	463	316 (100), 317 (50), 271 (15)	0.40%
Quinic acid	191	111 (100), 173 (20)	38.0%
Feruloylquinic acid	367	191 (100), 173 (5)	0.04%
Quercetin	301	179 (100), 151 (75), 273 (15), 257 (10)	0.03%
Tricin-O-glucoside	491	476 (100), 329 (70), 328 (15), 314 (5)	0.2%
Isorhamnetin-O-glucuronide	491	315 (100), 255 (10), 151 (5)	0.02%
	**[M + H]^+^ (*m*/*z*)**		
Laricitrin-O-glucoside	495	333 (100)	0.04%
Syringetin	347	291 (100), 153 (50), 287 (20)	0.08%
Kaempferol	287	153 (100), 121 (50), 213 (20), 229 (5)	0.1%
Apigenin	271	153 (100), 229 (30), 225 (30), 119 (10)	0.3%
Rutin	611	303 (100), 465 (30)	2.4%
Genistein	271	153 (100), 215 (85), 243 (60), 149 (25)	0.2%
Prunetin	285	229 (100), 257 (60), 267 (30), 163 (25)	1.2%
	**[M]^+^ (*m*/*z*)**		
Petunidin-O-pentoside	449	317 (100)	0.4%
Malvidin-O-pentoside	463	331 (100)	0.3%

## Supplementary Materials

The following are available online. Table S1: Values of total phenolic content (TPC), total flavonoid content (TFC) and antioxidant activities (DPPH and ABTS) in hydroalcoholic extract from asparagus hard-stem by-products, Table S2: Quantitative determination of free amino acids in asparagus edible-stem extract, Figure S1: LC-MS/MS ESI negative ion extracted chromatograms of identified compounds listed in Table 3, Figure S2: LC-MS/MS ESI positive ion extracted chromatograms of identified compounds listed in Table 3.
