# Supplementary material for: Anticancer Activity of Aqueous Extracts from Asparagus officinalis L. Byproduct on Breast Cancer Cells
Source: Molecules. 2021 Oct 21;26(21):6369. doi: 10.3390/molecules26216369 (PMC8588164; doi:10.3390/molecules26216369)
Supplement: Supplementary file 1 [file molecules-26-06369-s001.zip › molecules-1359343-supplementary.pdf]

molecules

Article

Anticancer activity of aqueous extract from *Asparagus officinalis L.* by-product on breast cancer cells

Arianna Romani [1,#], Fabio Casciano [1,2,#], Claudia Stevanin [3], Annalisa Maietti [3,4], Paola Tedeschi [3,4], Paola Secchiero [1], Nicola Marchetti [3,4,*] and Rebecca Voltan [1*]

1 Department of Translational Medicine and LTTA Centre, University of Ferrara, 44121 Ferrara, Italy
2 Interdepartmental Research Center for the Study of Multiple Sclerosis and Inflammatory and Degenerative Diseases of the Nervous System, University of Ferrara, 44121 Ferrara, Italy
3 Department of Chemistry, Farmaceutical and Agricultural Sciences, University of Ferrara, Italy
4 Terra&Acqua Tech Lab, Ferrara Technopole, Italy
# Co-first author
* Correspondence: nicola.marchetti@unife.it (NM); rebecca.voltan@unife.it (RV)

Table S1. Values of total phenolic content (TPC), total flavonoid content (TFC) and antioxidant activities (DPPH and ABTS) in hydroalcoholic extract from asparagus hard-stem by-products. Values are expressed in terms of gallic acid equivalents (GAE) and trolox equivalents (TE) per mg of dry matter (mg_{de}).

**TPC** ($\mu g_{GAE}/mg_{dm}$)	**276.1 ± 33.5**
**TFC** ($\mu g_{GAE}/mg_{dm}$)	231.4 ± 18.4
**DPPH** ($\mu mol_{TE}/mg_{dm}$)	0.271 ± 0.164
**ABTS** ($\mu mol_{TE}/mg_{dm}$)	0.426 ± 0.032

Table S2. Quantitative determination of free amino acids in asparagus edible-stem extract.

Amino acid	Concentration ($\mu g/mg_{de}$)
ALA	0.016 ± 0.019
ARG	0.234 ± 0.025
ASN	8.02 ± 0.94
ASP	0.379 ± 0.035
GLU	0.754 ± 0.033
HIS	0.135 ± 0.011
LEU+ILE	0.341 ± 0.024
LYS	17.54 ± 1.17
MET	0.211 ± 0.013
PHE	0.135 ± 0.017
PRO	1.463 ± 0.055
SER	1.159 ± 0.048
THR	0.223 ± 0.009
TRP	0.014 ± 0.005
TYR	0.054 ± 0.011
VAL	1.088 ± 0.031

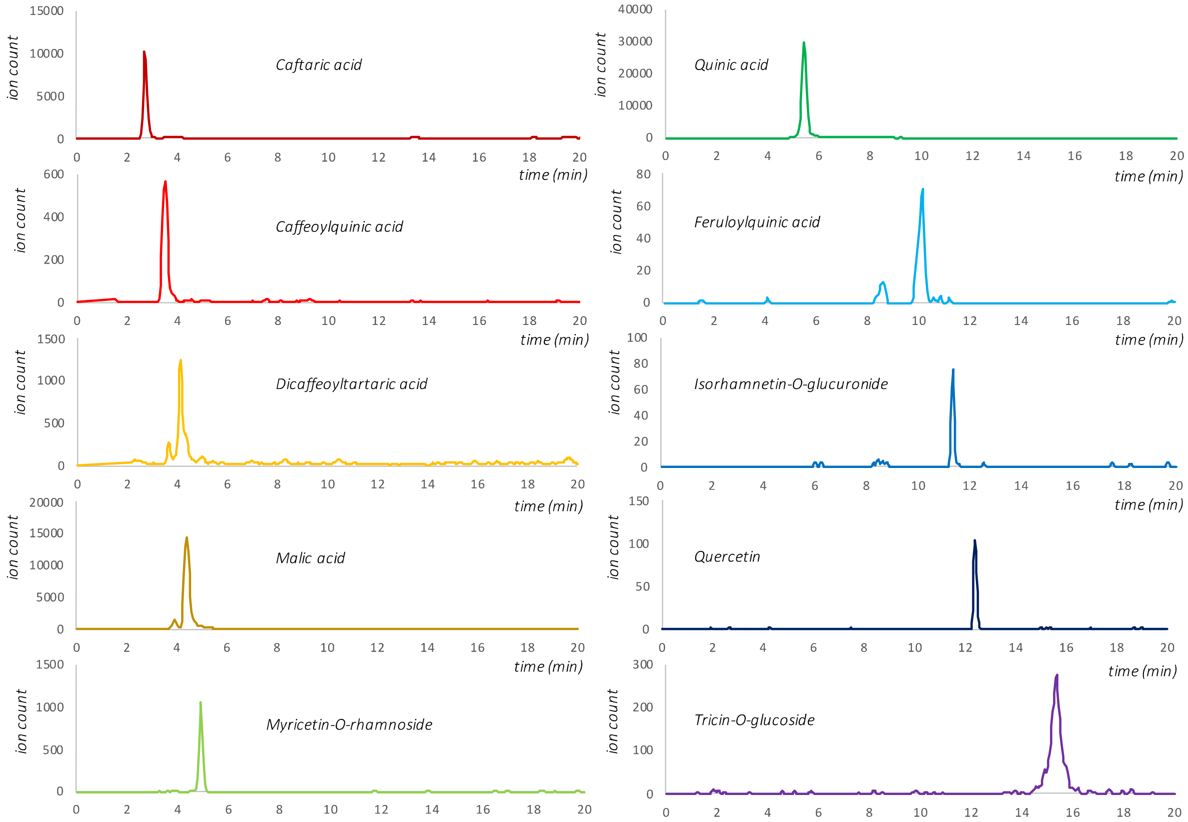

Figure S1. LC-MS/MS ESI negative ion extracted chromatograms of identified compounds listed in Table 3.

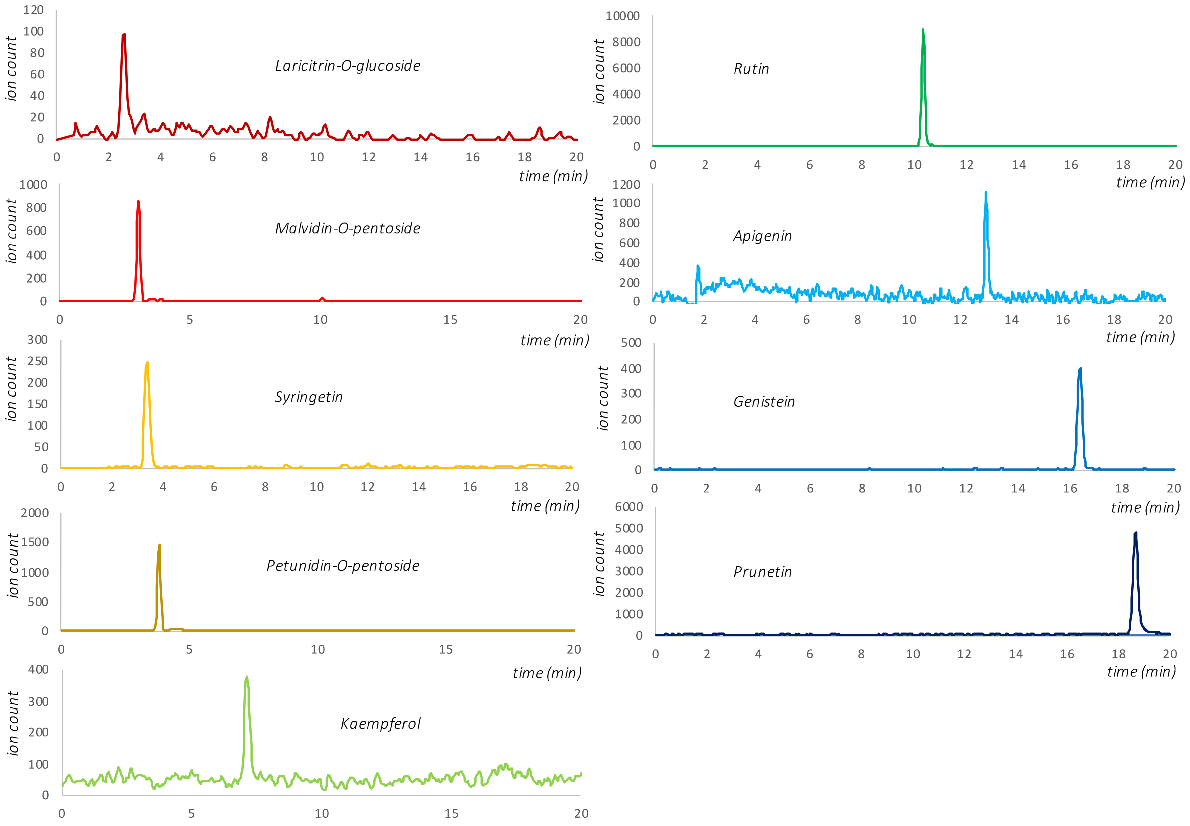

Figure S2. LC-MS/MS ESI positive ion extracted chromatograms of identified compounds listed in Table 3.
